# Analyses of the Mode of Action of an Alpha-Adrenoceptor Blocker in Substantia Gelatinosa Neurons in Rats

**DOI:** 10.3390/ijms22179636

**Published:** 2021-09-06

**Authors:** Daisuke Uta, Tsuyoshi Hattori, Megumu Yoshimura

**Affiliations:** 1 Department of Applied Pharmacology, Faculty of Pharmaceutical Sciences, University of Toyama, Toyama 930-0194, Japan; 2 Department of Medical Affairs, Asahi Kasei Pharma Corporation, Tokyo 100-0006, Japan; 3 Department of Orthopedic Surgery, Nakamura Hospital, Fukuoka 822-0002, Japan; yoshimura@onkokai.jp

**Keywords:** substantia gelatinosa, α1-adrenoceptor, naftopidil, noradrenaline, 5-HT, blind patch-clamp recording, micturition, inhibitory postsynaptic currents

## Abstract

To elucidate why naftopidil increases the frequency of spontaneous synaptic currents in only some substantia gelatinosa (SG) neurons, post-hoc analyses were performed. Blind patch-clamp recording was performed using slice preparations of SG neurons from the spinal cords of adult rats. Spontaneous inhibitory and excitatory postsynaptic currents (sIPSCs and sEPSCs, respectively) were recorded. The ratios of the frequency and amplitude of the sIPSCs and sEPSCs following the introduction of naftopidil compared with baseline, and after the application of naftopidil, serotonin (5-HT), and prazosin, compared with noradrenaline (NA) were evaluated. First, the sIPSC analysis indicated that SG neurons reached their full response ratio for NA at 50 μM. Second, they responded to 5-HT (50 μM) with a response ratio similar to that for NA, but prazosin (10 μM) did not change the sEPSCs and sIPSCs. Third, the highest concentration of naftopidil (100 μM) led to two types of response in the SG neurons, which corresponded with the reactions to 5-HT and prazosin. These results indicate that not all neurons were necessarily activated by naftopidil, and that the micturition reflex may be regulated in a sophisticated manner by inhibitory mechanisms in these interneurons.

## 1. Introduction

Life expectancy at birth is estimated to grow year on year according to a recent United Nations report [1]. The world’s population is growing older, with persons over the age of 65 being the fastest-growing age group. All countries anticipate an increase in the percentage of older people in their populations in the future. Aging, however, depresses physical activity, e.g., lower urinary tract function and impaired quality of life is observed in older people with lower urinary tract symptoms (LUTS), which comprise voiding and storage symptoms [2]. Voiding symptoms include slow stream, straining, and intermittency, especially in men with benign prostatic hyperplagia (BPH). Meanwhile, storage symptoms include daytime and nighttime frequency and urgency. Among these urinary symptoms, frequency and urgency are particularly serious for patients [3]. To manage LUTS with BPH, medications such as α1-adrenoceptor antagonists, 5α-reductase inhibitors, or phosphodiesterase-5 inhibitors are recommended [4]. α1-Adrenoceptor antagonists improve not only voiding symptoms but also storage symptoms, e.g., frequency and urgency [4,5]. The common mode of action for medications that improve voiding symptoms is smooth muscle relaxation in the urethra and prostate [6]. However, the mechanisms through which storage symptoms are alleviated are not well understood. Naftopidil and tamsulosin, which are α1-adrenoceptor antagonists widely used in Japan, have been shown to prolong the inter-contraction interval of the urinary bladder in rats according to a watershed experiment [7], improve micturition frequency in a murine cold-stress model [8], and increase the blood flow of microbe circulation in the urinary bladder with outlet obstruction in other murine models [9,10]. Moreover, naftopidil and tamsulosin increased bladder capacity, maximum desired volume, and initial desired volume according to a human pressure flow study [11]. Furthermore, the effect of naftopidil was found to be antagonized by intrathecal bicuculine and/or strychnine [12].

The micturition reflex is concordantly mediated by neural circuits in the brain, spinal cord, and peripheral ganglia [13]. The primary afferent nerves run from the urothelium to the spinal cord through dorsal root ganglia. Then, the signal transfers to the secondary neurons in the dorsal horn of the spinal cord. Electrophysiological recordings of neural activity in the spinal cord are useful to understanding the micturition reflex [14]. In patch-clamp recordings of substantia gelatinosa (SG) neurons in a slice preparation of an L6-S1 segment from a murine spinal cord, naftopidil increased the inhibitory postsynaptic current (IPSC) frequency, and this was antagonized by bicuculine or strychnine [15]. This effect of naftopidil was observed in only 38% of the SG neurons tested. Although the effect of naftopidil is steady in suppressing the micturition reflex in vivo, the reason why only some inhibitory SG neurons are excited by this drug remains unknown. In this study, we investigate the reasons why only some SG neurons respond to naftopidil.

## 2. Results

### 2.1. Characterization of SG Neurons for sIPSCs Responding to Noradrenalin

**Figure 1:**
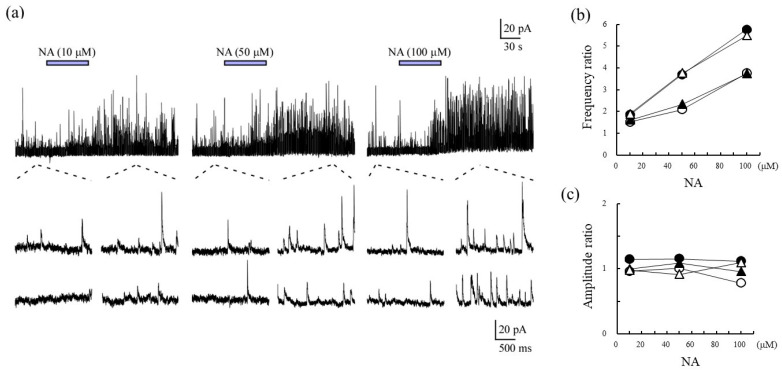
The effects of noradrenaline (NA; 10–100 μM) on spontaneous inhibitory postsynaptic currents (sIPSCs) in substantia gelatinosa (SG) neurons from the spinal cords of rats. A typical trace from a single SG neuron is presented (**a**). The ratios of frequency (**b**) and amplitude (**c**) were plotted against a series of concentrations for each SG neuron. The frequency/amplitude ratio represented the response before drug application compared with the response following application of the drug. The responses of each NG neuron are revealed as circles and triangles with open and closed. The same symbol means the same SG neuron.

Noradrenaline (NA) increased the frequency ratios of sIPSCs in the SG neurons in a concentration-dependent manner (Figure 1a,b) but had no effect on the amplitude ratios (Figure 1c). These results indicated that the responsiveness to NA in terms of the frequency of sIPSCs is concentration-dependent in SG neurons.

### 2.2. Correlation of sIPSCs Following Exposure of SGs to NA and Seratonin, Prazosin, and Naftopidil

**Figure 2:**
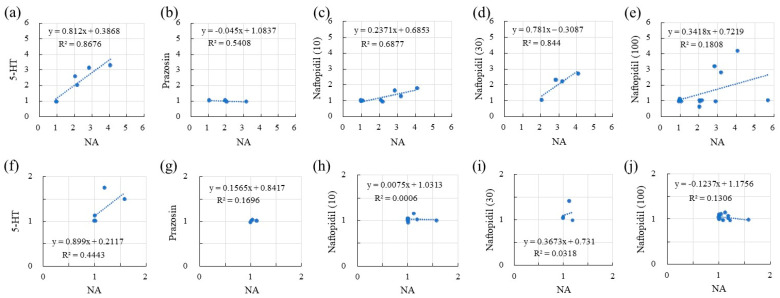
The association between NA (50 μM) and each ligand in terms of the response ratios of Table 1. HT (50 μM) (**a**), prazosin (10 μM) (**b**), and naftopidil (10, 30, and 100 μM) (**c**–**e**, respectively). Similarly, for amplitude, the associations with NA of 5-HT (50 μM) (**f**), prazosin (10 μM) (**g**), and naftopidil (s10, 30, and 100 μM) (**h**–**j**, respectively) are presented. The x and y axes reveal the ratio of frequency or amplitude of sIPSCs after adding the drugs (NA, 5-HT, prazosin, or naftopidil) to before the adding in the single SG neuron.

**Figure 3:**
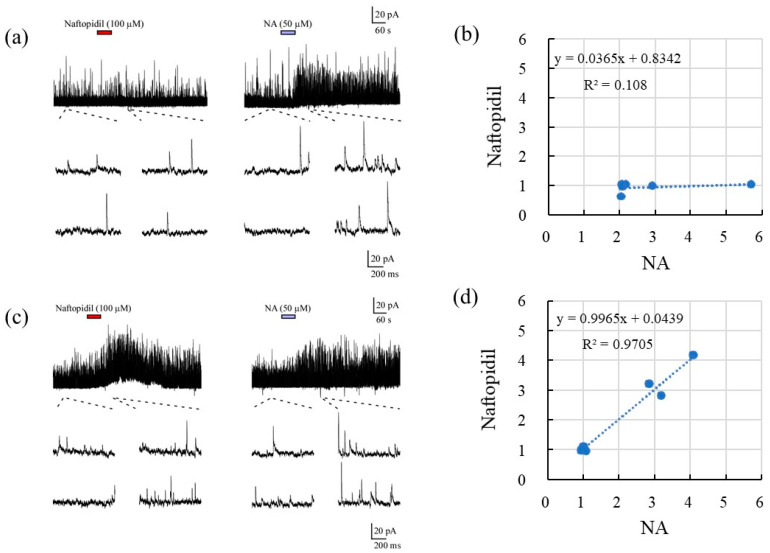
A stratified analysis of the sIPSCs for the frequency ratio response to NA (50 μM) and naftopidil (100 μM). The SG neurons exposed to naftopidil (100 μM) showed either a poor response (**a**,**b**) or a good response (**c**,**d**). Typical traces of both types are shown for a single SG neuron in (**a**,**c**), respectively. The associations of the response ratios between NA and naftopidil are plotted for each SG neuron (**b**,**d**). The x and y axes reveal the ratio of frequency or amplitude of sIPSCs after adding the drugs (NA, 5-HT, prazosin, or naftopidil) to before the adding in the single SG neuron.

The correlations between the sIPSCs associated with NA and serotonin (5-HT), prazosin, and naftopidil in terms of the frequency and amplitude of responses are summarized in Table 1 and Figure 2. With respect to frequency, 5-HT resulted in a fair correlation with NA (R, 0.931), with a regression line slope of 0.812. Although the application of prazosin resulted in a fair correlation with NA in terms of frequency (R, 0.735), the slope of the regression line was lower (−0.045). Naftopidil application showed excellent correlation with NA in a concentration-dependent manner at 10 and 30 μM (R, 0.829 and 0.919, respectively). However, the highest concentration (100 μM) reduced the correlation coefficient to 0.425. The regression line slopes indicated the same tendency. Additional analysis of the response to naftopidil at 100 μM indicated two regression line slopes that accorded with the prazosin-type (R, 0.108; slope 0.037) and 5-HT-type (R, 0.971; slope, 0.997) (Figure 3). In terms of amplitude (Table 1 and Figure 2), the response to 5-HT showed moderate correlation with that for NA (R, 0.667), and the value for the slope of the regression line was 0.899. Prazosin resulted in a weak correlation with the response to NA (R, 0.412) and low-level response as indicated by the low value for the slope (0.157). Naftopidil at 10, 30, and 100 μM resulted in a poor correlation with NA, resulting in correlation coefficients of 0.024, 0.178, and 0.361, respectively.

### 2.3. Morphological Analysis and sIPSCs

**Figure 4:**
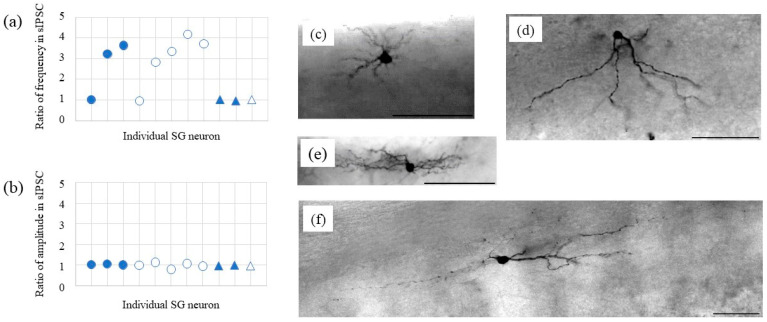
Responsiveness to naftopidil (100 μM) in terms of frequency (**a**) and amplitude (**b**) of sIPSCs with stratification by morphology of SG neurons. After recording the sIPSCs, dye was injected into the SG neurons. Then, the neurons were observed under a light microscope. Closed circles, radial; open circles, vertical; closed triangles, central; and open triangle, islet. Typical images of the SG neurons are shown: radial (**c**), vertical (**d**), central (**e**), and islet (**f**). Scale bars = 100 μm.

Following the electrophysiological recordings, we identified four types of SG neurons morphologically, i.e., radial (*n* = 3), vertical (*n* = 5), central (*n* = 2), and islet (*n* = 1) (Figure 4). Naftopidil increased the frequencies of the sIPSCs in the radial and vertical types of SG neurons but did not change the amplitudes of the sIPSCs.

### 2.4. Correlation of sEPSCs between NA and 5-HT, Prazosin, and Naftopidil

**Figure 5:**
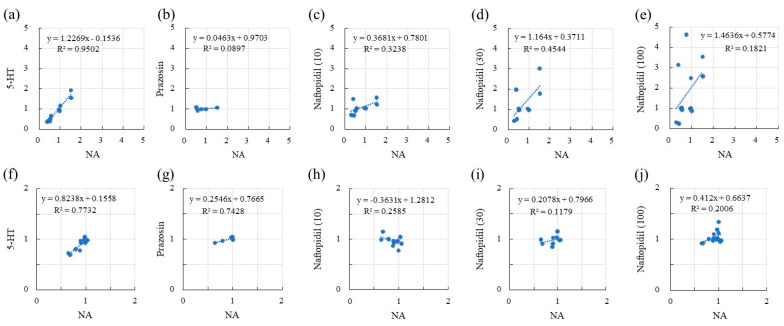
The association between NA (50 μM) and each ligand for the response ratios of sEPSCs in the SG neurons of spinal cords isolated from rats. Scatter plots for frequency were created for the association with NA of 5-HT (50 μM) (**a**), prazosin (10 μM) (**b**), and naftopidil (10, 30, and 100 μM) (**c**–**e**, respectively). Similarly, for amplitude, the associations between NA and 5-HT (50 μM) (**f**), prazosin (10 μM) (**g**), and naftopidil (10, 30, and 100 μM) (**h**–**j**, respectively) are presented. The x and y axes reveal the ratio of frequency or amplitude of sEPSCs after adding the drugs (NA, 5-HT, prazosin, or naftopidil) to before the adding in the single SG neuron. Each dot in the figures is shown as a response of the individual SG neuron.

The correlations of sEPSCs for the response ratios in terms of both frequency and amplitude between NA and 5-HT, prazosin, and naftopidil are summarized in Table 1 and Figure 5. In terms of frequency, 5-HT (50 μM) showed fair correlation with NA (50 μM) (R, 0.975), and the slope of the regression line was 1.227. Prazosin (10 μM) showed poor correlation with NA (R, 0.299), and the slope was independent to that for the frequency ratio of NA (0.049). Naftopidil (10–100 μM) showed a moderate correlation with NA (R, 0.427–0.674), while the slope increased from 0.368 to 1.464 in a concentration-dependent manner. With respect to amplitude, 5-HT also showed a fair correlation with NA (R, 0.879), and the slope of the regression line was 0.824. Prazosin showed a fair correlation with NA (R, 0.862), but the value for the slope of the regression line was low (0.255). Naftopidil showed moderate correlation with NA (R, 0.343–0.508). The slopes of the regression lines were inconsequential and small, i.e., lacking concentration dependency.

## 3. Discussion

In this study, we used patch-clamp recordings to measure sIPSCs and sEPSCs in SG neurons from the dorsal horns of spinal cords isolated from rats. The response ratios of the sIPSCs and sEPSCs before and after the addition of various drugs were calculated to represent the responsiveness of the SG neurons. We explored the mechanism of the low response rate to naftopidil of sIPSCs and sEPSCs by comparing it to the responsiveness to NA, 5-HT, and prazosin.

According to the frequency analysis of the sIPSCs, first, the SG neurons showed a change (1.0 to 5.7) in the response ratio for NA at 50 μM (Figure 2). Second, the frequency of sIPSCs in response to 5-HT (50 μM) corresponded with the response ratio to NA, but the responsiveness to prazosin (10 μM) was poor. Third, the highest concentration of naftopidil (100 μM) resulted in a two-way response, which corresponded with the response ratios seen for 5-HT (moderate) and prazosin (poor), respectively. NA, as a gold standard, facilitates the frequency of spontaneous synaptic currents and increases inhibition of sensory pathway. To elucidate why naftopidil increases the frequency of spontaneous synaptic currents in only some SG neurons, we checked response ratios for NA and naftopidil in the same SG neuron using 5-HT and prazosin as positive and negative control, respectively. We hypothesized that the group of some SG neurons shows full response to naftopidil as well as NA and 5-HT, but another group of other SG neurons does not. To demonstrate “full response”, we analysed correlations between NA and naftopidil. The value “around one” of correlation coefficient reveals likely consistent for response between NA and naftopidil. In the group of NOT response to naftopidil, the response ratio did not change for naftopidil regardless of the response ratio for NA in the same neuron as shown in prazosin. Therefore, the low response ratio to naftopidil, especially of sIPSCs, was elucidated from the low response to NA and low responsiveness to prazosin. In a previous report, the administration of naftopidil yielded an increase in the frequency of miniature (m)IPSCs in only 38% of SG neurons tested [15]. In addition, when postsynaptic sensitivity was evaluated using IPSCs, naftopidil produced outward IPSCs in 25% of the SG neurons tested, which were inhibited by bicuculine and strychnine [16]. These results suggest that the inhibitory effect of naftopidil is seen only in part of the neuronal population and mediates GABA/glycinergic neural transmission. To clarify whether a subclass of SG neurons relates to the low responsiveness to naftopidil, morphological observations of 11 SG neurons were made. Naftopidil increased the frequency of sIPSCs in two of three radial and four of five vertical types of SG neurons, and these were excitatory types of interneurons [17,18]. Therefore, we speculated that naftopidil acts on a synaptic terminal, which inputs to the radial and vertical types of SG neurons to inhibit excitation and induce sIPSCs. Although naftopidil did not affect central and islet SG neurons, a larger number of neurons should be tested to reach a plausible conclusion about each subclass.

With regard to the sEPSCs, the value of the slope for the regression line showing an association of the frequencies between NA and 5-HT was 1.227 (Table 1). This result indicated that the responsiveness of the SG neurons to NA and 5-HT was almost equivalent in terms of the frequency of the sEPSCs. The responsiveness of SG neurons to NA and naftopidil at 30 μM was also similar. Furthermore, the response to naftopidil was likely to be concentration-dependent, because the values for the slopes of the regression lines increased from 1.16 at 30 μM to 1.46 at 100 μM. Moreover, only three of nine and five of 13 SG neurons markedly responded to naftopidil at 30 and 100 μM, respectively. The low responsiveness to naftopidil of the sEPSCs may indicate that only a low number of SG neurons can respond to the drug. Low responsiveness of mEPSCs to α1-adrenoceptor antagonists was observed in a previous study using tamsulosin and silodosin [19,20]. Moreover, in a naftopidil characterization study observing EPSCs excited by dorsal root stimulation, naftopidil decreased both Aδ and C fiber-evoked EPSCs, while prazosin did not [21].

In an animal model, intrathecal naftopidil suppressed the micturition reflex, resulting in improved voiding frequency [7]. The effect of naftopidil was presumed to be mediated by sensory afferent C-fibers, because the effect was blocked by resiniferatoxin [22]. As these effects have been shown to be steady in animal experiments, the low response ratio seen for naftopidil in SG neurons in the slice patch-clamp recording may be totally sufficient. Recently, intrathecal administration of bicuculine and/or glycine was shown to antagonize the effect of intrathecal naftopidil on the prolongation of the inter-contraction interval of the urinary bladder in a rat cystometry study [12]. The study indicated that the GABA/glycine neuronal system participates in naftopidil’s effect on improving micturition frequency at the spinal cord level in vivo, similarly to the patch-clamp experiment [16]. Moreover, in our previous work [15,16,19,20,21] and in the present study, IPSCs and EPCSs were enhanced by naftopidil in some SG neurons. These results suggest that some inhibitory interneurons are excited, and those SG neurons that receive inhibitory inputs are activated.

A limitation is small number of SG neurons to be analyzed for morphology. Therefore, further study is needed with additional SG neurons in the morphological analysis to be able to make the hypothesis for neuron type plausible.

## 4. Materials and Methods

### 4.1. Animals and Slice Preparation

All experiments were performed in accordance with the “Guiding Principles for the Care and Use of Animals in the Field of Physiological Sciences” of the Physiological Society of Japan and were approved by the local Animal Experiment Committees of the University of Kyusyu and Kumamoto Health Science University. All efforts were made to minimize animal suffering and the number of animals used for the studies.

The methods for obtaining slices of adult rat spinal cord and blind patch-clamp recordings from SG neurons have been described in detail elsewhere [15]. Briefly, male adult Sprague Dawley rats (6–8 weeks of age, Kyudo, Fukuoka, Japan) were deeply anesthetized with urethane (1.2–1.5 g/kg, i.p.), and lumbosacral laminectomy was then performed. The lumbosacral spinal cord was removed and placed in a pre-oxygenated Krebs solution at 1–3 °C. Immediately after removal of the spinal cord, the rats were given an overdose of urethane and sacrificed by exsanguination. After removal of the dura mater, all ventral and dorsal roots were cut, and the pia-arachnoid membrane was then removed. The spinal cord was placed in a shallow groove formed in an agar block and glued to the bottom of the microslicer stage with cyanoacrylate adhesive. The spinal cord was immersed in cold Krebs solution, and a 500-μm-thick transverse or parasagittal slice was cut. The slice was placed on a nylon mesh in the recording chamber and then perfused at a rate of 10–15 mL/min with Krebs solution saturated with 95% O_2_ and 5% CO_2_ at 36 °C ± 1 °C. The Krebs solution contained the following: NaCl, 117 mM; KCl, 3.6 mM; CaCl_2_, 2.5 mM; MgCl_2_, 1.2 mM; NaH_2_PO_4_, 1.2 mM; NaHCO_3_, 25 mM; and glucose, 11 mM.

### 4.2. Blind Patch-Clamp Recording

Using transmitted illumination, the SG was easily discernible as a relatively translucent band across the dorsal horn in the transverse or parasagittal slice preparations. Blind whole-cell voltage-clamp recordings were made from SG neurons, as previously described [15]. The patch pipettes were filled with a solution containing the following: potassium gluconate solution (K-gluconate, 135 mM; KCl, 5 mM; CaCl_2_, 0.5 mM; MgCl_2_, 2 mM; EGTA, 5 mM; HEPES, 5 mM; and ATP-Mg, 5 mM; pH 7.2) or cesium solution (Cs_2_SO_4_, 110 mM; tetraethylammonium, 5 mM; CaCl_2_, 0.5 mM; MgCl_2_, 2 mM; EGTA, 5 mM; HEPES, 5 mM; and ATP-Mg, 5 mM; pH 7.2). The tip resistance of the patch pipettes was 6–12 MΩ. Series resistance was assessed according to the response to a 5-mV hyperpolarizing step. This value was monitored during the recording session, and data were rejected if the values changed by >15%. Signals were acquired with a patch-clamp amplifier (Axopatch 700A, Molecular Devices, Union City, CA, USA). The data were digitized with an analog-to-digital converter (Digidata 1321A, Molecular Devices), stored on a personal computer using a data acquisition program (Clampex version 9.0, Molecular Devices), and analyzed using a software package (Clampfit version 9.0, Molecular Devices). Cell recordings were made in voltage-clamp mode at holding potentials of −70 mV to record EPSCs and 0 mV for IPSCs. At these potentials, the GABA-/glycine-mediated IPSCs and glutamate-mediated EPSCs were negligible, respectively [15].

### 4.3. Morphological Analysis

The recorded neurons were anatomically identified post hoc as SG neurons by the intracellular injection of neurobiotin (0.2% in the pipette solution; Vector Laboratories, Burlingame, CA, USA). After electrophysiological recording, the spinal cord slices were fixed overnight in 4% paraformaldehyde in 0.1 M phosphate buffer (PB, pH 7.4) at 4 °C and then rinsed in PB. One or two neurons were recorded in each slice using the neurobiotin-containing pipette solution for post hoc morphological analysis. Neurobiotin-filled neurons were visualized using diaminobenzidine (DAB) staining histochemistry. For DAB staining, free-floating sections were incubated overnight in Vectastain (Elite kit; Vector Laboratory) with 0.1% Triton X-100. The peroxidase activity was revealed with DAB in the presence of hydrogen peroxide, and sections were mounted on gelatinized slides. According to the classification scheme proposed by previous studies [23,24], the neurobiotin-stained cells were classified into five types: vertical (*n* = 5, Figure 4c), radial (*n* = 3, Figure 4d), central (*n* = 2, Figure 4e), islet (*n* = 1, Figure 4f), and unclassified (not detected). Radial cells had dendrites that extended in several directions with roughly similar rostrocaudal and dorsoventral extents (Figure 4c). The dendritic trees of the islet cells were extremely elongated in the rostrocaudal direction (parallel to the layer borders) and limited in the dorsoventral and mediolateral directions (Figure 4f). Vertical cells were characterized by the pronounced ventral orientation of their dendritic tree (Figure 4d). Even if the rostrocaudal extension of the dendritic tree was in many cases still larger than the dorsoventral extension, the dendritic tree clearly descended into lamina II–IV. Distinguishing between islet and central cells is often difficult [25], although it has been reported that the rostrocaudal extent of islet cell dendritic trees is typically >400 μm and that axons of islet cells are generally limited to the volume of their dendritic trees (Figure 4e,f). Central cells displayed similar morphological characteristics to the islet cells but had a shorter rostrocaudal dendritic spread (Figure 4e).

### 4.4. Drug Application

Naftopidil (Asahi Kasei Pharma Corp., Tokyo, Japan) was dissolved in 1% dimethyl sulfoxide (DMSO) (Fujifilm Wako Pure Chemical Corp., Osaka, Japan) in Krebs solution. NA (Fujifilm Wako Pure Chemical Corp., Osaka, Japan), 5-HT (Sigma, St. Louis, MO, USA), and prazosin (Sigma, St. Louis, MO, USA) were dissolved in distilled water, and then diluted with Krebs solution. Each drug at each concentration was added into the bath after a washout period.

### 4.5. Statistical Analysis

We calculated the ratios for the frequency and amplitude of the response following the addition of drugs compared with the baseline. The values of the ratios were considered to represent responsiveness. Scatter plots for naftopidil, 5-HT, and prazosin compared with NA were created for the ratios of both frequency and amplitude. To investigate correlation, correlation coefficients and determination coefficients were calculated using Prism 8 (Graph Pad Software Inc., San Diego, CA, USA). The measurements for the correlation data were recorded from the same SG neuron to which a drug was added after an interval. To investigate responsiveness, the values of the slopes were generated from the regression lines between each drug and NA.

## 5. Conclusions

According to this study, not all SG neurons respond to naftopidil, and this may indicate that not all neurons are necessarily activated. Moreover, non-responding neurons may serve as backups in the case of disorder and injury or be involved in signal transduction outside of the voiding function. These mechanisms are considered to be appropriate actions. The micturition reflex may be regulated in a sophisticated manner by these inhibitory mechanisms in some interneurons. Further investigation is required to determine in greater detail how naftopidil suppresses the micturition reflex.

## Figures and Tables

**Table 1:** Correlation of sIPSCs and sEPSCs with noradrenalin.

Conc. (μM)	sIPSCs						sEPSCs					
	Frequency			Amplitude			Frequency			Amplitude		
	R	R^2^	Slope	R	R^2^	Slope	R	R^2^	Slope	R	R^2^	Slope
5-HT												
50	0.931	0.868	0.812	0.667	0.444	0.899	0.975	0.950	1.227	0.879	0.773	0.824
Prazosin												
10	0.735	0.541	−0.045	0.412	0.170	0.157	0.299	0.090	0.049	0.862	0.743	0.255
Naftopidil												
10	0.829	0.688	0.237	0.024	0.001	−0.008	0.569	0.324	0.368	0.508	0.259	−0.363
30	0.919	0.844	0.781	0.178	0.032	0.367	0.674	0.454	1.164	0.343	0.118	0.208
100	0.425	0.181	0.342	0.361	0.131	−0.124	0.427	0.182	1.464	0.448	0.201	0.412

Conc, concentration; sEPSCs, spontaneous excitatory postsynaptic currents; sIPSCs, spontaneous inhibitorypost synaptic currents; R, correlation coefficient; R^2^, determination coefficient; 5-HT, serotonin.

## Data Availability

The original contributions presented in the study are included in the article, further inquiries can be directed to the corresponding author.
